# Supplementary material for: Outcomes from Partner2Lose: a randomized controlled trial to evaluate 24-month weight loss in a partner-assisted intervention
Source: BMC Public Health. 2024 Jul 20;24:1948. doi: 10.1186/s12889-024-19464-z (PMC11265014; doi:10.1186/s12889-024-19464-z)
Supplement: Supplementary file 1 — Supplementary Material 1. [file 12889_2024_19464_MOESM1_ESM.docx]

Supplement 1. Model-estimated interdependence constructs for dietary change, differences, and associated CIs by treatment group and time

| Interdependence  Construct | Time point (months) | Participant-only estimated mean (95% CI), n | Partner-assisted  estimated mean (95% CI), n | Difference (95% CI) | p-value |
| --- | --- | --- | --- | --- | --- |
| Inclusion of other in self (possible range 1-7) | | | | | |
|  | Baseline | 4.1 (3.9, 4.3), 116 | 4.1 (3.9, 4.3), 115 | - | - |
|  | 3 | 3.9 (3.7, 4.2), 101 | 4.4 (4.1,4.6), 97 | -0.4 ( -0.7, -0.1) | 0.007 |
|  | 6 | 4.0 (3.8, 4.3), 107 | 4.3 (4.0, 4.5), 96 | -0.3 (-0.6, 0.0) | 0.090 |
|  | 9 | 4.2 (3.9, 4.4), 90 | 4.1 (3.8, 4.4), 75 | 0.0 (-0.3, 0.4) | 0.801 |
|  | 12 | 4.1 (3.8, 4.3), 101 | 4.2 (3.9, 4.4), 91 | -0.1 (-0.4, 0.2) | 0.588 |
|  | 15 | 4.0 (3.7, 4.3), 80 | 4.1 (3.8, 4.4), 76 | -0.1 (-0.4, 0.2) | 0.586 |
|  | 18 | 3.9 (3.7,4.2), 92 | 4.2 (3.9, 4.4), 81 | -0.2 (-0.5, 0.1) | 0.203 |
|  | 21 | 4.0 (3.8, 4.3), 79 | 4.1 (3.8, 4.4), 68 | -0.1, (-0.4, 0.3) | 0.690 |
| Couple efficacy (0-10) | | | | | |
|  | Baseline | 8.2 (7.9, 8.4), 116 | 8.2 (7.9, 8.4), 115 | - | - |
|  | 3 | 7.3 (6.9, 7.6), 101 | 7.7 (7.3, 8.0), 97 | -0.4 (-0.8, 0.0) | 0.048 |
|  | 6 | 7.3 (7.0, 7.7), 107 | 7.7 (7.4, 8.1), 96 | -0.4 (-0.8, 0.0) | 0.057 |
|  | 9 | 7.1 (6.7, 74), 90 | 7.8 (7.5, 8.2), 75 | -0.8 (-1.2, -0.3) | 0.001 |
|  | 12 | 7.1 (6.7, 74), 101 | 7.6 (7.3, 8.0), 91 | -0.5, (-0.9, -0.1) | 0.011 |
|  | 15 | 6.9 (6.5, 7.3), 80 | 7.2 (6.9, 7.6), 76 | -0.3 (-0.8, 0.1) | 0.146 |
|  | 18 | 6.9 (6.6, 7.3), 92 | 7.2 (6.9, 7.6), 81 | -0.3 ( -0.7, 0.1) | 0.140 |
|  | 21 | 6.6 (6.2, 7.0), 79 | 7.3 (6.9, 7.6), 68 | -0.7 (-1.1, -0.2) | 0.004 |
| Outcome efficacy (0-10) | | | | | |
|  | Baseline | 8.8 (8.6, 9.1), 116 | 8.8 (8.6, 9.1), 115 | - | - |
|  | 3 | 8.1 (7.8, 8.4), 101 | 8.1 (7.7, 8.4), 97 | 0.0 (-0.4, 0.4) | 0.931 |
|  | 6 | 8.0 (7.7, 8.3), 107 | 8.2 (7.9, 8.5), 96 | -0.2 (-0.6, 0.1) | 0.189 |
|  | 9 | 7.9 (7.6, 8.3), 90 | 8.2 (7.9, 8.5), 75 | -0.2 (-0.6, 0.2) | 0.262 |
|  | 12 | 7.8 (7.5, 8.1), 101 | 8.1 (7.8, 8.4), 91 | -0.3 (-0.7, 0.1) | 0.116 |
|  | 15 | 7.8 (7.5, 8.2), 80 | 7.8 (7.5, 8.2), 76 | 0.0 (-0.4, 0.4) | 0.974 |
|  | 18 | 7.7 (7.4, 8.0), 92 | 7.9 (7.5, 8.2), 81 | -0.2 (-0.6, 0.2) | 0.356 |
|  | 21 | 7.5 (7.1, 7.8), 79 | 7.8 (7.5, 8.2), 68 | -0.4 (-0.8, 0.1) | 0.099 |
| Communal coping (0-4) | | | | | |
|  | Baseline | 1.7 (1.6, 1.8), 116 | 1.7 (1.6, 1.8), 115 | - | - |
|  | 3 | 1.9 (1.7, 2.1), 101 | 2.0 (1.9, 2.2), 97 | -0.1 (-0.3, 0.0) | 0.132 |
|  | 6 | 1.9 (1.7, 2.0), 107 | 2.1 (1.9, 2.3), 96 | -0.2 (-0.4, 0.0) | 0.015 |
|  | 9 | 1.8 (1.7, 2.0), 90 | 2.0 (1.8, 2.2), 75 | -0.2 (-0.4, 0.0) | 0.081 |
|  | 12 | 1.7 (1.6, 1.9), 101 | 2.0 (1.9, 2.2), 91 | -0.3 (-0.5, -0.1) | 0.002 |
|  | 15 | 1.7 (1.6, 1.9), 80 | 2.0 (1.9, 2.2), 76 | -0.3 (-0.5, -0.1) | 0.006 |
|  | 18 | 1.7 (1.6, 1.9), 92 | 2.0 (1.8, 2.2), 81 | -0.3 (-0.5, -0.1) | 0.008 |
|  | 21 | 1.7 (1.6, 1.9), 79 | 1.8 (1.6, 2.0), 68 | -0.1 (-0.3, 0.1) | 0.519 |
| Social support (1-5) | | | | | |
|  | Baseline | 3.1 (3.0, 3.2), 116 | 3.1 (3.0, 3.2), 115 | - | - |
|  | 3 | 3.2 (3.1, 3.3). 101 | 3.5 (3.2, 3.6), 97 | -0.3 (-0.4, -0.1) | 0.001 |
|  | 6 | 3.1 (3.0, 3.3), 107 | 3.5 (3.3, 3.6), 96 | -0.3 (-0.5, -0.2) | <0.001 |
|  | 9 | 3.2 (3.1, 3.4), 90 | 3.5 (3.3, 3.7), 75 | -0.3 (-0.4, -0.1.) | 0.002 |
|  | 12 | 3.2 (3.1, 3.3), 101 | 3.4 (3.3, 3.5), 92 | -0.2 (-0.4, 0.0) | 0.011 |
|  | 15 | 3.2 (3.0, 3.3), 80 | 3.4 (3.2, 3.5), 76 | -0.2 (-0.4, -0.1) | 0.007 |
|  | 18 | 3.1 (3.0, 3.3), 92 | 3.3 (3.2, 3.5), 81 | -0.2 ( -0.4, 0.0) | 0.019 |
|  | 21 | 3.1 (2.9, 3.2), 79 | 3.3 (3.1, 3.4), 68 | -0.2, (-0.3, 0.0) | 0.067 |
| Sabotage (1-5) | | | | | |
|  | Baseline | 2.6 (2.4, 2.7), 116 | 2.6 (2.4, 2.7), 115 | - | - |
|  | 3 | 2.5 (2.3, 2.6), 101 | 2.3 (2.2, 2.5), 97 | 0.2 (0.0, 0.3) | 0.062 |
|  | 6 | 2.4 (2.3, 2.6), 107 | 2.3 (2.1, 2.5), 96 | 0.1 (-0.1, 0.3) | 0.232 |
|  | 9 | 2.5 (2.3, 2.6), 90 | 2.3 (2.2, 2.5), 75 | 0.1 ( -0.1, 0.3) | 0.157 |
|  | 12 | 2.4 (2.3, 2.6), 101 | 2.4 (2.2, 2.5), 91 | 0.1 (-0.1, 0.3) | 0.356 |
|  | 15 | 2.4 (2.2, 2.5), 80 | 2.5 (2.3, 2.6), 76 | -0.1 ( -0.3, 0.1) | 0.425 |
|  | 18 | 2.5 (2.4, 2.7), 92 | 2.4 (2.2, 2.6), 81 | 0.1 (-0.1, 0.3) | 0.249 |
|  | 21 | 2.6 (2.5, 2.8), 79 | 2.5 (2.3, 2.7), 68 | 0.1 (-0.1, 0.3) | 0.282 |
