# Supplementary material for: Outcomes from Partner2Lose: a randomized controlled trial to evaluate 24-month weight loss in a partner-assisted intervention
Source: BMC Public Health. 2024 Jul 20;24:1948. doi: 10.1186/s12889-024-19464-z (PMC11265014; doi:10.1186/s12889-024-19464-z)
Supplement: Supplementary file 2 — Supplementary Material 2. [file 12889_2024_19464_MOESM2_ESM.docx]

Supplement 2. Model-estimated interdependence constructs for physical activity, differences, and associated CIs by treatment group and time

| Interdependence Construct | Time point (months) | Participant-only estimated mean (95% CI), n | Partner-assisted  estimated mean (95% CI), n | Difference (95% CI) | p-value |
| --- | --- | --- | --- | --- | --- |
| Inclusion of other in self (possible range 1-7) | | | | | |
|  | Baseline | 3.4 (3.2, 3.6), 116 | 3.4 (3.2, 3.6), 115 | - | - |
|  | 3 | 3.4 (3.2, 3.7), 101 | 3.4 (3.1, 3.7), 97 | 0.1 (-0.3, 0.4) | 0.692 |
|  | 6 | 3.4 (3.2, 3.7), 107 | 3.4 (3.1, 3.6), 96 | 0.1 (-0.2, 0.4) | 0.659 |
|  | 9 | 3.5 (3.2, 3.8), 90 | 3.3 (3.0, 3.6), 75 | 0.2 (-0.2, 0.5) | 0.324 |
|  | 12 | 3.5 (3.3, 3.8), 101 | 3.5 (3.2, 3.8), 91 | 0.0 (-0.3, 0.3) | 0.854 |
|  | 15 | 3.6 (3.3, 3.8), 80 | 3.4 (3.1, 3.7), 76 | 0.2 (-0.2, 0.5) | 0.282 |
|  | 18 | 3.4 (3.2, .7), 92 | 3.4 (3.1, 3.7), 81 | 0.0 (-0.3, 0.4) | 0.812 |
|  | 21 | 3.6 (3.3, 3.9), 79 | 3.2 (2.9, 3.5), 68 | 0.4 (0.0, 0.7) | 0.047 |
| Couple efficacy (0-10) | | | | | |
|  | Baseline | 8.0 (7.6, 8.3), 116 | 8.0 (7.6, 8.3), 115 | - | - |
|  | 3 | 6.9 (6.5, 7.3), 101 | 7.1 (6.7, 7.5), 97 | -0.2 (-0.7, 0.2) | 0.327 |
|  | 6 | 7.0 (6.6, 7.4), 107 | 7.0 (6.6, 7.4), 96 | 0.0 (-0.5, 0.4) | 0.958 |
|  | 9 | 7.0 (6.6, 7.4), 90 | 7.1 (6.7, 7.5), 75 | -0.2 (-0.7, 0.3) | 0.520 |
|  | 12 | 6.9 (6.5, 7.3), 101 | 7.0 (6.6, 7.4), 91 | -0.1 (-0.6. 0.4) | 0.663 |
|  | 15 | 6.8 (6.3, 7.2), 80 | 6.8 (6.4, 7.3), 76 | -0.1 (-0.6, 0.4) | 0.724 |
|  | 18 | 6.5 (6.1, 6.9), 92 | 6.6 (6.2, 7.0), 81 | -0.1 (-0.6, 0.4) | 0.616 |
|  | 21 | 6.5 (6.1, 6.9), 79 | 6.7 (6.2, 7.1), 68 | -0.2 (-0.7, 0.3) | 0.493 |
| Outcome efficacy (0-10) | | | | | |
|  | Baseline | 8.4 (8.1, 8.6), 116 | 8.4 (8.1, 8.6), 115 | - | - |
|  | 3 | 7.3 (6.9, 7.7), 101 | 7.4 (7.0, 7.7), 97 | -0.1 (-0.5, 0.4) | 0.803 |
|  | 6 | 7.3 (7.0,7.7), 107 | 7.4 (7.0, 7.7), 96 | 0.0 (-0.5, 0.4) | 0.883 |
|  | 9 | 7.5 (7.2, 7.9), 90 | 7.5 (7.1, 7.9), 75 | 0.1 (-0.4, 0.5) | 0.800 |
|  | 12 | 7.4 (7.1, 7.8), 101 | 7.2 (6.8, 7.6), 91 | 0.2 (-0.2, 0.7) | 0.321 |
|  | 15 | 7.3 (6.9, 7.7), 80 | 7.2 (6.8, 7.6), 76 | 0.1 (-0.4, 0.6) | 0.738 |
|  | 18 | 7.1 (6.8, 7.5), 92 | 7.1 (6.7, 7.5), 81 | 0.0 (-0.4, 0.5) | 0.868 |
|  | 21 | 7.1 (6.8, 7.5), 79 | 7.1 (6.7, 7.5), 68 | 0.1 (-0.4, 0.6) | 0.823 |
| Communal coping (0-4) | | | | | |
|  | Baseline | 1.5 (1.3, 1.6), 116 | 1.5 (1.3, 1.6), 115 | - | - |
|  | 3 | 1.6 (1.4, 1.8), 101 | 1.7 (1.5, 1.9), 97 | -0.1 (-0.3, 0.1) | 0.384 |
|  | 6 | 1.6 (1.4, 1.8), 107 | 1.7 (1.6, 1.9), 96 | -0.1 (-0.3, 0.1) | 0.238 |
|  | 9 | 1.7 (1.5, 1.9), 90 | 1.7 (1.5, 1.9), 75 | 0.0 (-0.2, 0.2) | 0.984 |
|  | 12 | 1.7 (1.6, 1.9), 101 | 1.7 (1.6, 1.9), 91 | 0.0 (-0.2, 0.2) | 0.997 |
|  | 15 | 1.6 (1.4, 1.8), 80 | 1.7 (1.5, 1.9), 76 | 0.0 (-0.3, 0.2) | 0.668 |
|  | 18 | 1.6 (1.4, 1.7), 92 | 1.7 (1.5, 1.9), 81 | -0.1 (-0.3, 0.1) | 0.322 |
|  | 21 | 1.6 (1.5, 1.8), 79 | 1.6 (1.4, 1.8), 68 | 0.1 (-0.2, 0.3) | 0.589 |
| Social support (1-5) | | | | | |
|  | Baseline | 2.9 (2.8, 3.0), 116 | 2.9 (2.8, 3.0), 115 | - | - |
|  | 3 | 2.9 (2.7, 3.0), 101 | 3.1 (2.9, 3.2), 97 | -0.2 (-0.4, 0.0) | 0.017 |
|  | 6 | 2.9 (2.8, 3.1), 107 | 3.0 (2.9, 3.2), 96 | -0.1 (-0.3, 0.1) | 0.315 |
|  | 9 | 3.0 (2.8, ,3.1), 90 | 3.0 (2.9, 3.2) 75 | -0.1 (-0.3, 0.1) | 0.371 |
|  | 12 | 2.9 (2.8, 3.1), 101 | 3.1 (3.0, 3.3), 91 | -0.2 (-0.4, 0.0) | 0.026 |
|  | 15 | 2.9 (2.7, 3.1), 80 | 3.1 (2.9, 3.2), 76 | -0.2 (-0.3, 0.0) | 0.109 |
|  | 18 | 2.9 (2.7, 3.1), 92 | 3.0 (2.8, 3.1), 81 | -0.1 (-0.3, 0.1) | 0.387 |
|  | 21 | 2.8 (2.7, 3.0), 79 | 2.9 (2.8, 3.1), 68 | -0.1 (-0.3, 0.1) | 0.407 |
| Sabotage (1-5) | | | | | |
|  | Baseline | 2.6 (2.5, 2.7), 116 | 2.6 (2.5, 2.7), 115 | - | - |
|  | 3 | 2.6 (2.5, 2.8), 101 | 2.5 (2.4, 2.7), 97 | 0.1 (-0.1, 0.2) | 0.455 |
|  | 6 | 2.5 (2.4, 2.7), 107 | 2.4 (2.3, 2.6), 96 | 0.1 (-0.1, 0.3) | 0.356 |
|  | 9 | 2.5 (2.4, 2.7), 90 | 2.6 (2.5, 2.8), 75 | -0.1 (-0.3, 0.1) | 0.315 |
|  | 12 | 2.6 (2.5, 2.8), 101 | 2.6 (2.4, 2.7), 91 | 0.1 (-0.1, 0.2) | 0.549 |
|  | 15 | 2.6 (2.5, 2.8), 80 | 2.6 (2.5, 2.8), 76 | 0.1 (-0.1, 0.3) | 0.545 |
|  | 18 | 2.7 (2.5, 2.8), 92 | 2.6 (2.5, 2.8), 81 | 0.1 (-0.1, 0.2) | 0.505 |
|  | 21 | 2.6 (2.4, 2.7), 79 | 2.7 (2.6, 2.9), 68 | -0.1 (-0.3, 0.1) | 0.199 |
